# Phylogenetic analyses of distantly related clades of bent-toed geckos (genus *Cyrtodactylus*) reveal an unprecedented amount of cryptic diversity in northern and western Thailand

**DOI:** 10.1038/s41598-020-70640-8

**Published:** 2021-01-27

**Authors:** Siriwadee Chomdej, Waranee Pradit, Chatmongkon Suwannapoom, Parinya Pawangkhanant, Korakot Nganvongpanit, Nikolay A. Poyarkov, Jing Che, Yangchun Gao, Shiping Gong

**Affiliations:** 1 Department of Biology, Faculty of Science, Chiang Mai University, Chiang Mai, 50200 Thailand; 2 Research Center in Bioresources for Agriculture, Industry and Medicine, Chiang Mai University, Chiang Mai, 50200 Thailand; 3 School of Agriculture and Natural Resources, University of Phayao, Phayao, 56000 Thailand; 4 Department of Veterinary Bioscience and Veterinary Public Health, Faculty of Veterinary Medicine, Chiang Mai University, Chiang Mai, 50100 Thailand; 5 Excellence Center in Veterinary Bioscience, Chiang Mai University, Chiang Mai, 50200 Thailand; 6 Biological Faculty, Department of Vertebrate Zoology, Moscow State University, Moscow, Russia 119234; 7 Laboratory of Tropical Ecology, Joint Russian-Vietnamese Tropical Research and Technological Center, Hanoi, Vietnam; 8 State Key Laboratory of Genetic Resources and Evolution, Kunming Institute of Zoology, Chinese Academy of Sciences, Kunming, 650223 Yunnan China; 9 Southeast Asia Biodiversity Research Institute, Chinese Academy of Sciences, Yezin, Nay Pyi Taw, 05282 Myanmar; 10 Guangdong Key Laboratory of Animal Conservation and Resource Utilization, Guangdong Public Laboratory of Wild Animal Conservation and Utilization, Guangdong Institute of Applied Biological Resources, Guangdong Academy of Science, Guangzhou, 510260 Guangdong China

**Keywords:** Phylogenetics, Biogeography

## Abstract

*Cyrtodactylus* species are the most diverse of the geckos and are widely distributed in Southeast Asia, including Thailand. However, their patterns of distribution, especially in northern and western parts of Thailand, remain unknown because few *Cyrtodactylus* species in these regions have been described. Thus, a data set of mitochondrial NADH dehydrogenase 2 (*ND2*) gene and flanking tRNAs from *Cyrtodactylus* found in northern and western Thailand, including contiguous areas, was assembled to elucidate phylogenetic relationships and identify the distribution patterns of these geckos. The results showed four well-supported clades, a northwestern clade (A), a northern clade (B), a western clade (C), and a special clade characterized by specific morphological features (D). Clades A–C were grouped with strong support by the geography of their localities from northern Thailand (Mae Hong Son and Chiang Mai Provinces) along the Tenasserim mountain ranges to Phang-Nga Province, Thailand. Clade D is a distinct clade of *Cyrtodactylus* species characterized by a tuberculate and prehensile tail and distributed widely in mainland Southeast Asia. Overall, the results suggest a pattern of geographic separation and distribution of *Cyrtodactylus* in northern and western Thailand. Additionally, this study provides evidence of a hidden biodiversity of *Cyrtodactylus* in these regions.

## Introduction

Reptiles have a long, complex evolutionary history that has produced diverse species distributed throughout the world, both in water and on land^1^. Since reptiles play important roles in natural systems as predators, preys, and seed dispersers as well as serving as bioindicators of the health of the environment^2–4^, the conservation of reptiles is critical. Accurate species delimitation is therefore necessary to understand their speciation, diversification, and biogeography for conservation purposes. However, this remains a challenge due to the existence of cryptic species and species complexes. This may be a result of the long and complex evolution of reptiles, which first appeared on Earth more than 250 million years ago^1,5–7^. Among the Gekkonids (geckos), which is a group of lizards (Sauria), the most diverse group of reptiles in the world, the taxonomy of numerous species, including *Hemidactylus*^8–10^, *Hemiphyllodactylus*^11–13^, and *Cyrtodactylus*^14–16^, is still controversial.

The Gekkonidae found in Thailand are highly diverse, and include approximately 90 species. Among these species, 38 species of bent-toed geckos (*Cyrtodactylus*) are the most well-documented and show wide distribution in Thailand^17^. The bent-toed geckos are Asian geckos that are widely distributed on the Indian subcontinent and radiated eastward through Indochina and AustraloPapua^18^. They are divided to two major groups depending on whether they inhabit forests or limestone caves. As they are part of complex ecosystems harboring syntopic and microendemic amphibians and reptiles^19^, changes of these unique or restricted ecosystems strongly affect the survival of these geckos. Although a high number of *Cyrtodactylus* species have already been discovered (approximately 290 species have been described to date) and the discovery of many more is predicted based on the rapid annual discovery rate of new *Cyrtodactylus* species^17^, several are defined as endemic, including *C. sanook*, *C. saiyok*, and *C. phuketensis*^20–22^. Endemic species are at higher risk of extinction because their habitat is limited or unique. Additionally, it has been reported in recent years that the rate of destruction of *Cyrtodactylus* habitat has increased due to both forest land encroachment and wildfires, which occur most year, especially in Southeast Asia^23^ where *Cyrtodactylus* is primarily distributed.

**Figure 1:**
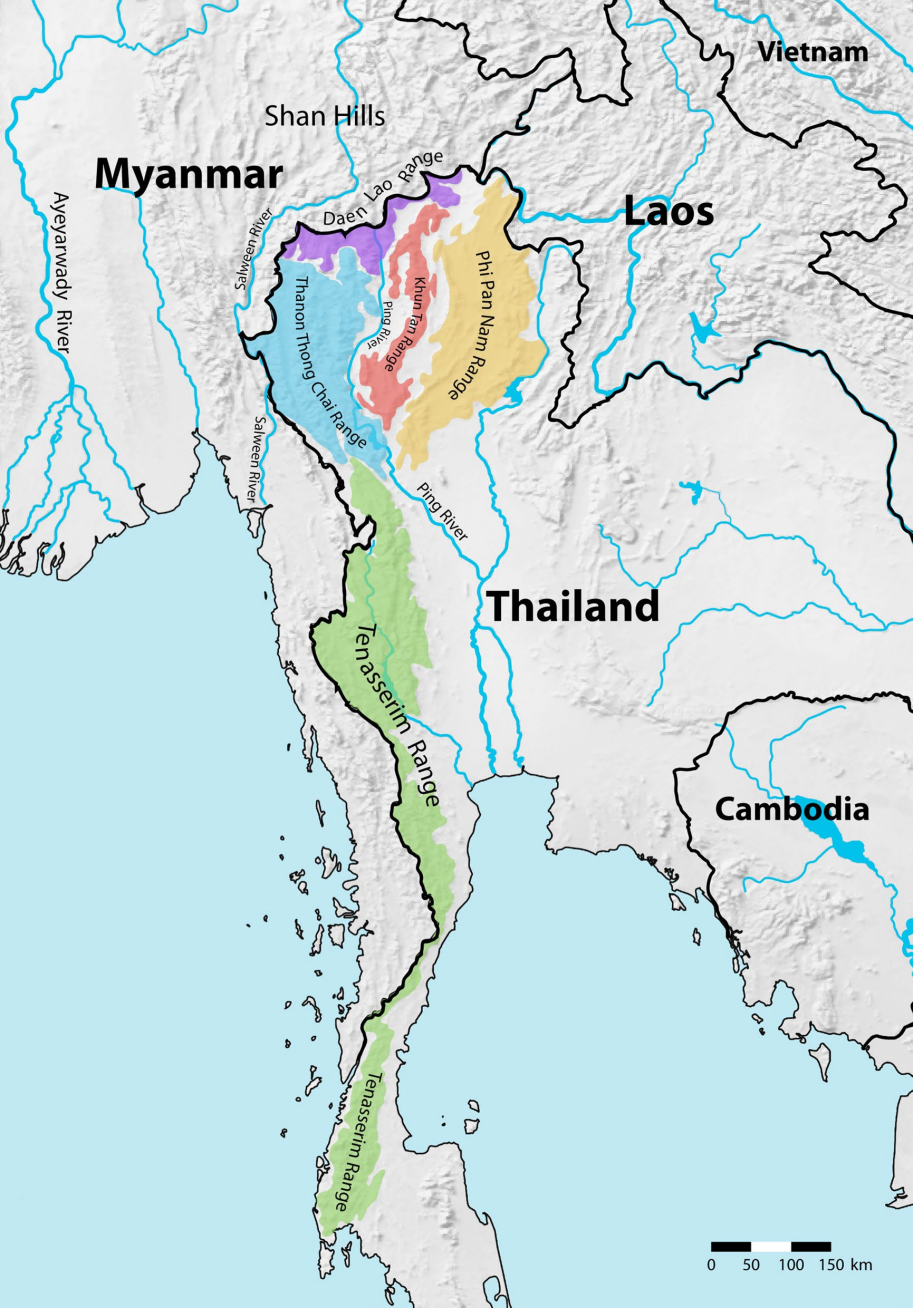
Geography of northern and western Thailand (www.simplemappr.net).

Despite the fact that they provide suitable habitat for bent-toed geckos, many areas in northern and western Thailand (Fig. 1) with mountainous landscapes and karsts have not been surveyed. Additionally, mountain ranges in the northern and western areas show a similar pattern to those in some parts of southern Thailand where many species of bent-toed geckos have previously been found^14,15,21,22,24–26^. Although, a few studies of *Cyrtodactylus* species in northern Thailand have been performed, nevertheless molecular phylogenetic analysis was not included in these works^27–32^. Several species of *Cyrtodactylus* have recently been described as cryptic species both in Thailand (*C. pulchellus* complex^14^) and elsewhere (*C. irregularis*^16^ and *C. peguensis*^15^). This indicates that widespread taxa commonly comprise complex species that are morphologically similar but genetically divergent. Thus, in the present study, molecular tools were utilized to reveal the phylogenetic relationships and geographic distribution of *Cyrtodactylus* species in northern and western Thailand based on mitochondrial data. The results provide insights into the range of distribution of *Cyrtodactylus* in northern and western Thailand that will aid in the conservation of endemic bent-toed geckos.

## Results

### Sequence characteristics and divergence

In this study, multiple pairs of primers were used to amplify approximately 1500 bp of mitochondrial *ND2* gene with flanking tRNAs. All new sequences were deposited in the GenBank database (Accession numbers: MT468895–MT468896, MT468898–MT468912, and MT497800–MT497807). After alignment of the new sequences and additional sequences from GenBank, 1325 sites were found to be variable and 1338 were found to be parsimony-informative. Nucleotide frequencies were as follows: A = 31.87%, T = 20.50%, C = 34.07%, and G = 13.54% (all analyses excluded outgroups).

The uncorrected p-distances of *Cyrtodactylus* in northern and western Thailand, including surrounding areas and species closely related to geckos in this region, were calculated and are shown in Supplementary Table S1. The mean pairwise sequence divergence among *Cyrtodactylus* species analyzed in this study was 23%. The intraspecific sequence divergence ranged from 0.00 to 8.27%. The greatest sequence divergence was observed in *C. tigroides*. The mean interspecific p-distance ranged from 0.94% (between *C. doisuthep* and *Cyrtodactylus* sp. 5 from Inthanon National Park, Chiang Mai) to 36.44% (between *C. sinyineensis* and *C. elok*).

### Phylogenetic relationships

**Figure 2:**
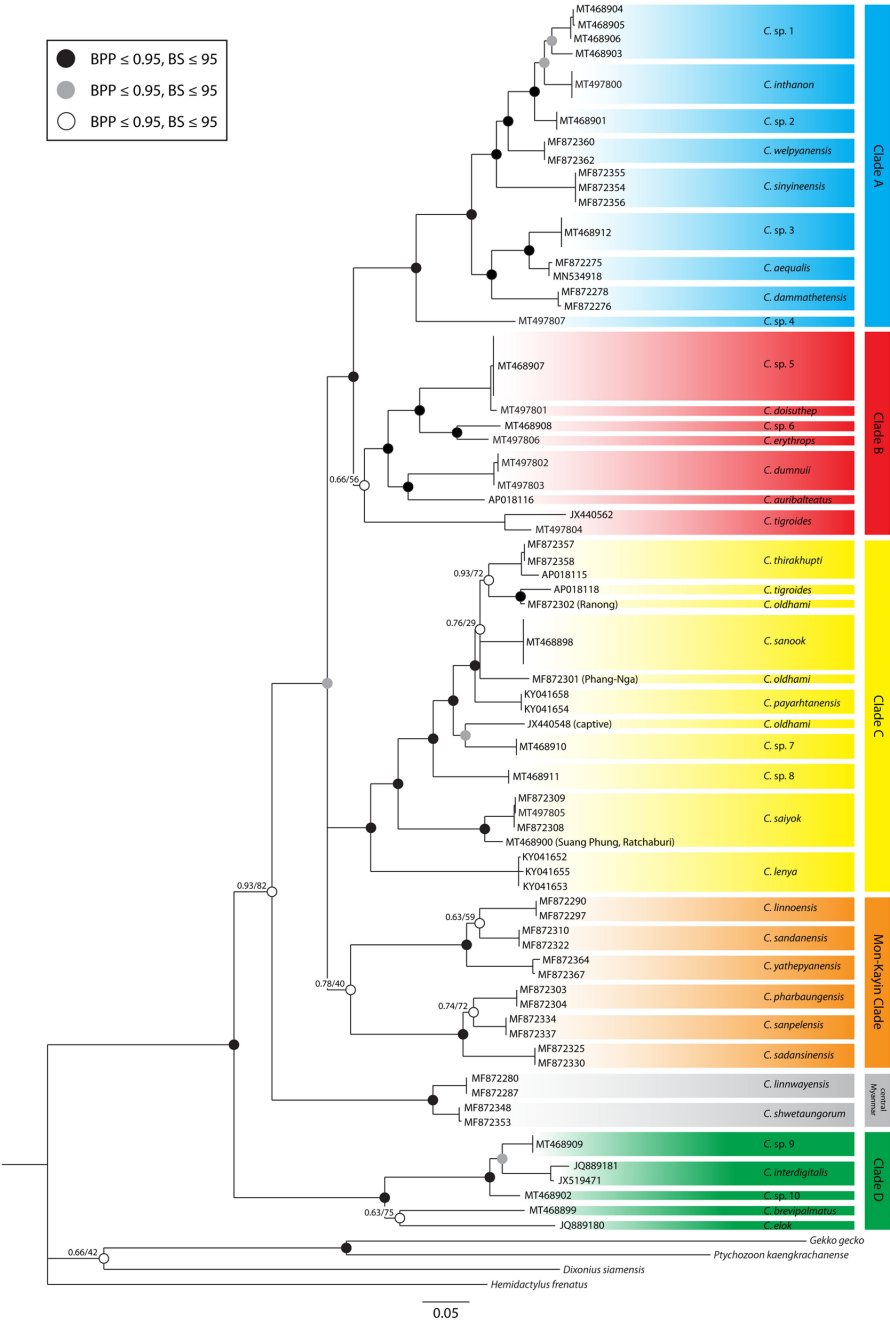
Phylogenetic relationship among geckos in the genus *Cyrtodactylus* distributed in northern and western Thailand based on the mitochondrial *ND2* gene with flanking tRNA sequences. Values near the nodes represented Bayesian posterior probability (BPP) and ML bootstrap support values (BS, 1,000 replicates) as BPP and BS, respectively.

The final Bayesian inference (BI) and maximum likelihood (ML) trees had similar topologies with slightly differences only at several poorly supported nodes. Based on the sequences of the *ND2* gene and flanking tRNAs of *Cyrtodactylus* distributed in northern and western Thailand, the phylogenetic tree showed four *Cyrtodactylus* clades, designated clades A–D (Fig. 2). Clades A–C were sister taxa, but the clade D was not closely related to the other *Cyrtodactylus* taxa. Clade A comprised samples collected from Chiang Mai Province, Tak Province, and Kamphaeng Phet Province in northern and western Thailand, including some *Cyrtodactylus* species found in Kayin State (*C. welpyanensis* and *C. sinyineensis*) and Mon State (*C. aequalis* and *C. dammathetensis*), Myanmar, which neighbor Tak Province at the same latitudinal range. Clade A was found to be as most closely related to clade B and comprised specimens from Chiang Mai Province, Mae Hong Son Province, and Phitsanulok Province, northern Thailand. Clade C included specimens found in Kanchanaburi Province, Ratchaburi Province, Ranong Province, Chumporn Province, Phang-Nga Province, and Surat Thani Province, western and southern Thailand, including *C. lenya* and *C. payarhtanensis* from Lenya National Park, Myanmar. This clade was also the most complex of the sister clades, especially in terms of *C. oldhami* from Ranong Province, which was shown to be closely related to *C. tigroides* (AP018118) or the unidentified *Cyrtodactylus* sp. 7 from Kanchanaburi Province. Additionally, *Cyrtodactylus* species from nearby locations (Kayin State and Mon State, Myanmar) represented a sister taxon to clade C. For *C. linnwayensis* and *C. shwetaungorum*, which were found in Mandalay Region and Shan Region, central Myanmar, respectively, there was no close relationship to clade A–C or the Kayin–Mon clade. Clade D showed, with strong support (BPP/BS = 1.00/99), diverse distribution in Thailand, and included *C. interdigitalis* which distributed in Loei Province, Thailand and some parts of Laos, the unidentified *Cyrtodactylus* sp. 9 and *Cyrtodactylus* sp. 10 from western Thailand (Tak Province and Kanchanaburi Province, respectively), *C. brevipalmatus* from Nakhon Si Thammarat Province, and *C. elok* from Narathiwat Province and Malaysia. This reveals syntopy between this clade and *Cyrtodactylus* in other clades (clades A and C). In Tak Province, two *Cyrtodactylus* species, *Cyrtodactylus* sp. 9 and *Cyrtodactylus* sp. 1, were found in the same location. Similarly, *Cyrtodactylus* sp. 10 and *Cyrtodactylus* sp. 7 were found at the same sampling site in Kanchanaburi Province.

## Discussion

In this study, the phylogenetic trees were constructed based on DNA sequences of *ND2* gene and flanking tRNAs. The topology of the trees revealed four clades of *Cyrtodactylus* in northern and western Thailand: a northwestern clade (A), a northern clade (B), a western clade (C), and a special clade (D) found in many parts of Thailand and its neighbors, Laos, Myanmar, and Malaysia. Clades A–C support the strong geographic segregation of *Cyrtodactylus* in mainland Southeast Asia^18^ and the divergence of *Cyrtodactylus* in this region, as represented by the previously unknown *Cyrtodactylus* species discovered in this study. Additionally, this result is in accordance with the distribution of *Cyrtodactylus* in Southeast Asia, in which *Cyrtodactylus* from Thailand belong to a central Indochina clade^18^. Clade D is a special clade in which geckos are characterized by their tuberculate and prehensile tails.

**Figure 3:**
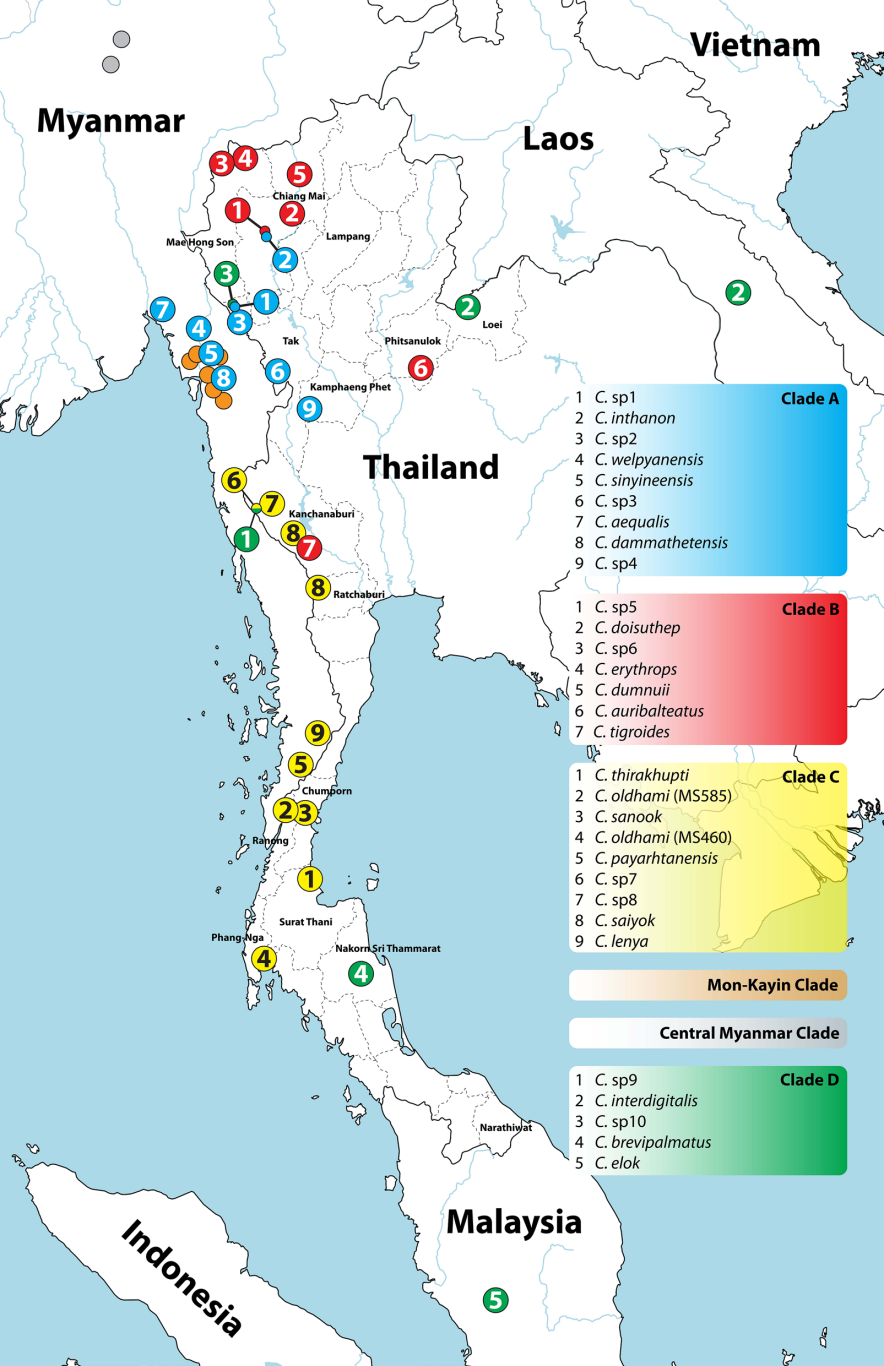
Distribution of *Cyrtodactylus* species found in northern and western Thailand. The map was downloaded from https://www.simplemappr.net.

As we found in our analysis, *Cyrtodactylus* species in the northwestern clade (clade A) are closely related to those previously described from Mon State and Kayin state, Myanmar, which are categorized as the “sinyineensis group”^19^. At the basal node of this clade, two lineages were recovered, the first large lineage is closely related to the sinyineensis group, while the other comprise only one member. The large lineage showed that *Cyrtodactylus* was distributed in the Salween Basin and nearby areas, including Inthanon Mountain. Since *Cyrtodactylus* sp. 1, sp. 2, and sp. 3 from Tak Province were found in the basin of Moei River, which is a tributary of the Salween River (Fig. 3), it is unsurprising that these new species are closely related to *Cyrtodactylus* in the sinyineensis group from the Salween Basin in Myanmar. Furthermore, *C. inthanon* from Tiger Head Mountain in Inthanon National Park, Chiang Mai Province seems to be more closely related to *Cyrtodactylus* species in clade B than those in clade A because *C. inthanon* inhabitats near the Ping Basin. This raises the possibility that some historical event influenced the embedding of *C. inthanon* in clade A, such as a significant change in climate, a change in sea levels, or a change in the direction of river flow that severely fragmented the habitat of this species. Regarding the second lineage, our results strongly support the segregation of *Cyrtodactylus* sp. 4, found in Mae Wong National Park, Kampaeng Phet Province, into a monophyletic lineage, which is in agreement with its locality on the east side of the northern Tenasserim Range, adjacent to the Ping Basin. This suggests that the Tenasserim Range may be a geographic barrier for both lineages. However, due to a lack of samples from this region on the east side of the Tenasserim Range, the distribution range of the second lineage of this clade remain unknown.

In the northern clade (clade B), a close relationship among *Cyrtodactylus* species from northern Thailand was represented. This clade also revealed strong geographic segregation based on the mountain ranges in this region. The samples from the Thanon Thong Chai Range (*Cyrtodactylus* sp. 5 and *C. doisuthep* from Chiang Mai Province) and the Daen Lao Range (*Cyrtodactylus* sp. 6 and *C. erythrops* from Mae Hong Son Province) were more closely related than the samples from the Khun Tan Range (*C. dumnuii* from Chiang Mai Province) and Thung Salaeng Luang National Park (*C. auribalteatus* from Phitsanulok Province) (Fig. 3). This leads us to hypothesize about a separate lineage of *C. khelangensis* which found in Lampang Province and located in the Phi Pan Nam Range. Interestingly, we noticed a relatively close relationship between *Cyrtodactylus* sp. 5 and *C. doisuthep* with a p-distance of 0.94%. This low divergence suggests that these species are in fact the same. They should be compared morphologically to further investigate this interesting relationship. Given that the number of known *Cyrtodactylus* species is low considering the amount of suitable biogeographic habitat in northern Thailand, there are likely many undescribed species hidden in this region. As shown by the present results, *C. tigroides* collected from Kanchanaburi Province were placed in this clade, albeit with weak support (BPP/BS = 0.66/56). Nevertheless, we noticed that genetic divergence among *C. tigroides* from nearby collection sites (Saiyok District, Kanchanaburi Province) was relatively high (8.27%). This suggests the existence of a *C. tigroides* species complex. Additional analysis using a combination of morphological and molecular data is thus necessary for this species.

While members of *Cyrtodactylus* in clade C have been described and analyzed for some time^20,21,33–35^, the complexity of *Cyrtodactylus* species within this clade has only been revealed recently^19^. This clade seems to be the most relative to the others in this study. As shown in the phylogenetic trees, *C. tigroides* (AP018118) with unknown locality was sister to *C. oldhami* (MF872302) from Ranong Province. Captive *C. oldhami* (JX440548) was also closely related to *Cyrtodactylus* sp. 7 from Kanchanaburi Province, and *C. oldhami* (MF872301) from Phang-Nga Province, and was separated into a single lineage. A high divergence (p-distance) ranging from 8.30 to 12.16% was observed among three distinct *C. oldhami* individuals. Similarly, *C. saiyok* collected in this study from Suan Phueng District, Ratchaburi Province (MT468900) exhibited relatively high divergence (5.15%) with the other *C. saiyok* from Kanchanaburi Province and Suan Phueng District, Ratchaburi Province (from a previous study). This suggests a species complex of *C. oldhami, C. tigroides*, and *C. saiyok*, and therefore the need to determine the morphological characteristics of these species. This complexity and the wide distribution range of *Cyrtodactylus* in this clade along the Tenasserim Range from Kanchanaburi Province to Phang-Nga Province indicate that intensive study of *Cyrtodactylus* in this region should be performed. The main reason for the uncertainty surrounding *Cyrtodactylus* in clade C is a lack of important information on habitats and molecular data on many species, both known and unknown. Without this information, relationships and distribution patterns of *Cyrtodactylus* in this clade will remain unclear.

Bayesian inference strongly supported the close relationships between *Cyrtodactylus* from the Salween Basin in Mon and Kayin States, Myanmar (Mon–Kayin clade) and the yathepyanensis and sadansineensis groups of *Cyrtodactylus* in clades A, B, and C. For the central Myanmar clade, *Cyrtodactylus* of the linnwayensis group from the Shan Hills were also in a separate clade. The topology of the tree was based on their geographic differences, in accordance with a previous study^19^. The species in these clades were included in the present analysis because the Salween Basin shares a border with northern and western regions of Thailand. Moreover, many new species of *Cyrtodactylus* from the Salween Basin are still being described^36,37^.

An exclusive clade (clade D) containing *Cyrtodactylus* that widely distribute in Thailand (western, southern, and northeastern parts) and neighboring countries (Laos, Myanmar, and Malaysia)^38–40^ was strongly supported by BI and ML analyses (BPP/BS = 1.0/98). When considered in terms of geography, the radiation and relationships of *Cyrtodactylus* in this clade did not correlate. On the other hand, a strong correlation was observed between the topology of *Cyrtodactylus* in clade D and the presence of a tuberculate and prehensile tail. This supports the phylogenetic trees previously constructed by Grismer et al.^41^ and Harvey et al.^38^. Two major lineages occur in the prehensile tail group. The first lineage (1) contained *Cyrtodactylus* with digital webbing and a caudal fringe (*C. brevipalmatus, C. elok*, and *C. interdigitalis*). The second lineage (2) comprised *Cyrtodactylus* with spinose tubercles in the ventrolateral fold of the body (*C. spinosus, C. stresemanni, C. nuaula, C. serratus, C. lateralis*, and *C. durio*)^38^. Although the prehensile tail group was categorized based on morphology in the present study, Harvey et al.^38^ found that this special group could be grouped by geography. The first lineage was found on mainland Southeast Asia and is referred to as the mainland Southeast Asian clade, while the second was found on peninsulas and islands in Southeast Asia and is referred to as the insular clade. In the present study, two previously undescribed *Cyrtodactylus* species from Tak Province and Kanchanaburi Province were added to the analysis. The results showed that they were sister taxa to *C. interdigitalis* (Fig. 2). This also suggests the extended radiation in mainland Southeast Asia of *Cyrtodactylus* species with prehensile tail.

## Conclusion

The phylogeny presented in this study illustrates the relationship and distribution of *Cyrtodactylus* species in northern and western Thailand. Four clades of *Cyrtodactylus* were recovered: a northwestern clade (clade A), a northern clade (clade B), a western clade (clade C), and a special clade (clade D). Clade A distributed from the Salween Basin in Myanmar to the Thanon Thong Chai mountain range in northern Thailand and was grouped with the “sinyineensis group”. Clade B was found to disperse in major mountain ranges of northern Thailand, including the Thanon Thong Chai, Daen Lao, and Khun Tan ranges. Clade C radiated along the Tenasserim Range from Kanchanaburi Province to Phang-Nga Province. Clade D was unique among the four clades in that *Cyrtodactylus* in this clade were grouped based on their tuberculate and prehensile tails, and were distributed widely in mainland Southeast Asia. The present phylogeny will be a useful guide for the identification or description of many unknown *Cyrtodactylus* species, especially from northern and western Thailand. The results also raise the possibility of analysis of other aspects of *Cyrtodactylus* species, such as their evolution, ecomorphology, and phylogenetic endemism. Finally, this study implies a hidden biodiversity of *Cyrtodactylus* in northern and western Thailand.

## Materials and methods

### Taxon sampling

Eighty-eight individual samples of *Cyrtodactylus* with seven outgroup samples were collected by hands from 19 localities in Thailand. All samples were euthanized with ether, dissected to obtain liver tissues, and stored in absolute ethanol. Animal care and use for research purposes were conducted with permission and guideline approved by the Institutional Ethical Committee of Animal Experimentation of the University of Phayao, Phayao, Thailand (certificate number UP-AE61-01-04-0022 issued to Chatmongkon Suwannapoom) and complied strictly with the ethical conditions of the Thailand Animal Welfare Act. Field work, including the collection of animals in the field and specimen exportation, was authorized by the Institute of Animals for Scientific Purpose Development (IAD), Bangkok, Thailand (permit number U1-04995-2559 issued to Siriwadee Chomdej). This research was permitted by the Department of National Parks, Wildlife and Plant Conservation (DNP).

### DNA extraction and amplification

All 95 samples were subsampled to 41 samples including *Cyrtodactylus* and outgroups based on their taxa and localities. Genomic DNA was extracted from the samples using a DNA extraction kit (Omega Bio-Tek) following the manufacturer’s protocol. The mitochondrial NADH dehydrogenase subunit 2 (*ND2*) gene with partial flanking tRNA regions was amplified to obtain sequences of approximately 1500 bp. The selected regions were amplified using *Taq* DNA polymerase in a total volume of 25 μl with the following thermal cycler profile: 95 °C for 5 min, followed by 35 cycles of 95 °C for 30 s, 50 °C for 30 s, 72 °C for 1 min, and a final extension step at 72 °C for 5 min. PCR products were visualized by 1.5% agarose gel electrophoresis under a UV illuminator. The amplified products were subsequently purified using NucleoSpin Gel and PCR Clean-up (Macherey–Nagel) and sequenced in both directions by A T G C (Thailand) using the same primer pairs as in the amplification procedure (Table 1).

**Table 1:** Lists of primers used for PCR amplification and sequencing.

Primer name	Sequence (5′–3′)	References
L4437	AAGCTTTCGGGCCCATACC	Macey et al.^42^
L4437b	AAGCAGTTGGGCCCATACC	Macey et al.^43^
H5934	AGRGTGCCAATGTCTTTGTGRTT	Macey et al.^42^
H5937a	GTGCCAATGTCTTTGTG	Macey et al.^42^
H5937b	AGGGTTCCGATATCTTTRTG	Macey et al.^44^

### Phylogeny

Forty-one new mitochondrial sequences obtained in this study and additional sequences retrieved from GenBank were used to create a phylogenetic tree using Bayesian inference (BI) and maximum likelihood (ML) methods. Homologous sequences of related species in the genus *Cyrtodactylus*, and of outgroups (*Gekko gecko*, *Ptychozoon kaengkrachanense*, *Dixonius siamensis,* and *Hemidactylus frenatus*) were downloaded from GenBank (Supplementary Table S2)^14,18,19,37,53–57^. New sequences were checked and assembled by AutoSeqMan^45^. All sequences were aligned using MUSCLE 3.6^46^, checked by eye for accuracy, and trimmed to minimize missing characters in MEGA v10.0.5^47^. Phylogenies were reconstructed using ML and BI methods. jModelTest 2.1.7^48^ was used to select an appropriate nucleotide substitution model for BI. The GTR + G model were chosen as the best-fit model following the Bayesian information criterion^49^ for the *ND2* gene and flanking tRNAs. BI analysis was performed by the CIPRES web server^50^. For BI analyses, the Monte Carlo Markov chain length was run for 10,000,000 generations and sampled every 1000 generations with a burn-in of 25%. Convergence was assessed based on the average standard deviation of split frequencies (below 0.01) and ESS values (over 200) in Tracer 1.7^51^. ML analyses were performed using RAxML with 1,000 bootstrap replicates using the rapid bootstrap feature (random seed value 12,345)^52^. In addition to phylogenetic tree–based methods, we calculated row pairwise sequence divergence using uncorrected p-distances in MEGA v10.0.5^47^.

## Supplementary information

Supplementary file1

Supplementary file2

## Data Availability

The dataset generated and/or analyzed during current study are available within the published article and its supplementary information files.
