# Supplementary material for: Phylogenetic analyses of distantly related clades of bent-toed geckos (genus Cyrtodactylus) reveal an unprecedented amount of cryptic diversity in northern and western Thailand
Source: Sci Rep. 2021 Jan 27;11:2328. doi: 10.1038/s41598-020-70640-8 (PMC7840752; doi:10.1038/s41598-020-70640-8)
Supplement: Supplementary file 1 — Supplementary file1 [file 41598_2020_70640_MOESM1_ESM.pdf]

Supplementary information
Table S1

Phylogenetic analyses of distantly related clades of bent-toed geckos (genus *Cyrtodactylus*) reveal an unprecedented amount of cryptic diversity in northern and western Thailand

Siriwadee Chomdej[1,2]*, Waranee Pradit[1], Chatmongkon Suwannapoom[3], Parinya Pawangkhanant[3], Korakot Nganvongpanit[4,5], Nikolay A. Poyarkov[6,7], Jing Che[8,9], Yangchun Gao[10], Shiping Gong[10]*

Table S1 Interspecific uncorrected p-distance (percentage) based on the sequences of *ND2* gene (below the diagonal line) and intraspecific p-distance (on the diagonal line) of *Cyrtodactylus* species in phylogenetic analysis.

	**Species**	**outgroup**	**1**	**2**	**3**	**4**	**5**	**6**	**7**	**8**	**9**	**10**	**11**	**12**	**13**	**14**	**15**	**16**	**17**	**18**	**19**	**20**
	outgroups	0.56																				
**1**	*Cyrtodactylus inthanon*	0.52	0.00																			
**2**	*Cyrtodactylus* sp5	0.58	0.23	0.00																		
**3**	*Cyrtodactylus sanook*	0.53	0.24	0.24	0.00																	
**4**	*Cyrtodactylus brevipalmatus*	0.54	0.31	0.31	0.31	-																
**5**	*Cyrtodactylus saiyok* (Suan Phueng)	0.54	0.24	0.24	0.18	0.31	-															
**6**	*Cyrtodactylus saiyok*	0.55	0.24	0.25	0.18	0.31	0.05	0.00														
**7**	*Cyrtodactylus* sp. 2	0.53	0.05	0.21	0.25	0.31	0.24	0.25	0.00													
**8**	*Cyrtodactylus* sp. 10	0.53	0.31	0.32	0.30	0.19	0.29	0.30	0.30	-												
**9**	*Cyrtodactylus* sp. 1	0.52	0.05	0.22	0.25	0.31	0.25	0.24	0.05	0.30	0.02											
**10**	*Cyrtodactylus* sp. 3	0.52	0.13	0.25	0.27	0.31	0.29	0.28	0.13	0.31	0.13	0.00										
**11**	*Cyrtodactylus* sp. 6	0.52	0.21	0.14	0.25	0.29	0.22	0.23	0.21	0.29	0.22	0.20	-									
**12**	*Cyrtodactylus* sp. 9	0.53	0.31	0.33	0.32	0.21	0.30	0.31	0.30	0.07	0.31	0.32	0.30	0.00								
**13**	*Cyrtodactylus* sp. 7	0.52	0.23	0.23	0.10	0.30	0.16	0.17	0.22	0.28	0.23	0.25	0.22	0.30	0.00							
**14**	*Cyrtodactylus* sp. 8	0.50	0.22	0.22	0.13	0.30	0.16	0.17	0.21	0.29	0.22	0.23	0.20	0.30	0.12	0.01						
**15**	*Cyrtodactylus doisuthep*	0.53	0.20	0.01	0.23	0.30	0.23	0.23	0.20	0.30	0.21	0.23	0.13	0.31	0.21	0.20	-					
**16**	*Cyrtodactylus dumnuii*	0.50	0.20	0.18	0.23	0.30	0.22	0.23	0.18	0.31	0.20	0.21	0.17	0.32	0.20	0.21	0.16	0.00				
**17**	*Cyrtodactylus tigroides*	0.52	0.23	0.24	0.25	0.32	0.25	0.26	0.23	0.30	0.23	0.23	0.21	0.31	0.24	0.23	0.22	0.21	0.08			
**18**	*Cyrtodactylus erythrops*	0.51	0.21	0.14	0.25	0.29	0.22	0.23	0.20	0.30	0.21	0.21	0.07	0.31	0.22	0.20	0.12	0.16	0.21	-		
**19**	*Cyrtodactylus* sp. 4	0.52	0.17	0.22	0.26	0.32	0.25	0.24	0.17	0.31	0.18	0.18	0.21	0.31	0.23	0.23	0.20	0.21	0.23	0.20	-	
**20**	*Cyrtodactylus welpyanensis*	0.53	0.09	0.23	0.25	0.31	0.26	0.27	0.07	0.30	0.08	0.13	0.22	0.30	0.24	0.22	0.21	0.18	0.22	0.21	0.17	0.00
**21**	*Cyrtodactylus sineyineensis*	0.54	0.12	0.24	0.26	0.33	0.27	0.28	0.11	0.30	0.12	0.15	0.22	0.32	0.23	0.23	0.22	0.20	0.25	0.23	0.19	0.10
**22**	*Cyrtodactylus dammathetensis*	0.51	0.14	0.24	0.28	0.31	0.28	0.29	0.13	0.30	0.13	0.12	0.20	0.30	0.26	0.24	0.22	0.21	0.22	0.20	0.17	0.13
**23**	*Cyrtodactylus aequalis*	0.54	0.13	0.24	0.27	0.32	0.28	0.28	0.13	0.31	0.13	0.06	0.20	0.32	0.25	0.23	0.22	0.19	0.24	0.19	0.17	0.12
**24**	*Cyrtodactylus aurtibalteatus*	0.50	0.20	0.16	0.22	0.29	0.23	0.23	0.21	0.29	0.21	0.22	0.16	0.29	0.19	0.20	0.15	0.14	0.21	0.16	0.20	0.19

Table S1 (Continued) Interspecific uncorrected p-distance (percentage) based on the sequences of *ND2* gene (below the diagonal line) and intraspecific p-distance (on the diagonal line) of *Cyrtodactylus* species in phylogenetic analysis.

	**Species**	**21**	**22**	**23**	**24**	**25**	**26**	**27**	**28**	**29**	**30**	**31**	**32**	**33**	**34**	**35**	**36**	**37**	**38**	**39**	**40**	**41**
	outgroups																					
**1**	*Cyrtodactylus inthanon*																					
**2**	*Cyrtodactylus* sp. 5																					
**3**	*Cyrtodactylus sanook*																					
**4**	*Cyrtodactylus brevipalmatus*																					
**5**	*Cyrtodactylus saiyok* (Suan Phueng)																					
**6**	*Cyrtodactylus saiyok*																					
**7**	*Cyrtodactylus* sp. 2																					
**8**	*Cyrtodactylus* sp. 10																					
**9**	*Cyrtodactylus* sp. 1																					
**10**	*Cyrtodactylus* sp. 3																					
**11**	*Cyrtodactylus* sp. 6																					
**12**	*Cyrtodactylus* sp. 9																					
**13**	*Cyrtodactylus* sp. 7																					
**14**	*Cyrtodactylus* sp. 8																					
**15**	*Cyrtodactylus doisuthep*																					
**16**	*Cyrtodactylus dumnuii*																					
**17**	*Cyrtodactylus tigroides*																					
**18**	*Cyrtodactylus erythrops*																					
**19**	*Cyrtodactylus* sp. 4																					
**20**	*Cyrtodactylus welpyanensis*																					
**21**	*Cyrtodactylus sineyineensis*	0.00																				
**22**	*Cyrtodactylus dammathetensis*	0.16	0.00																			
**23**	*Cyrtodactylus aequalis*	0.15	0.11	0.00																		
**24**	*Cyrtodactylus aurtibalteatus*	0.22	0.21	0.21	-																	

Table S1 (Continued) Interspecific uncorrected p-distance (percentage) based on the sequences of *ND2* gene (below the diagonal line) and intraspecific p-distance (on the diagonal line) of *Cyrtodactylus* species in phylogenetic analysis.

	**Species**	**outgroup**	**1**	**2**	**3**	**4**	**5**	**6**	**7**	**8**	**9**	**10**	**11**	**12**	**13**	**14**	**15**	**16**	**17**	**18**	**19**	**20**
**25**	*Cyrtodactylus tigroides* (AP018116)	0.56	0.27	0.28	0.10	0.34	0.18	0.19	0.27	0.33	0.27	0.28	0.26	0.33	0.13	0.15	0.25	0.25	0.28	0.25	0.27	0.27
**26**	*Cyrtodactylus lenya*	0.53	0.25	0.25	0.20	0.31	0.20	0.19	0.25	0.30	0.25	0.28	0.22	0.30	0.20	0.18	0.23	0.23	0.25	0.22	0.25	0.24
**27**	*Cyrtodactylus oldhami* (captive)	0.52	0.24	0.25	0.12	0.33	0.16	0.17	0.24	0.30	0.25	0.25	0.25	0.31	0.10	0.14	0.24	0.24	0.25	0.24	0.24	0.25
**28**	*Cyrtodactylus oldhami* (Phang-Nga)	0.52	0.24	0.26	0.08	0.33	0.17	0.20	0.24	0.30	0.24	0.28	0.24	0.31	0.10	0.13	0.23	0.24	0.25	0.23	0.24	0.26
**29**	*Cyrtodactylus oldhami* (Ranong)	0.52	0.24	0.24	0.08	0.32	0.16	0.18	0.23	0.30	0.24	0.25	0.23	0.30	0.11	0.12	0.22	0.23	0.24	0.22	0.24	0.23
**30**	*Cyrtodactylus payarhtanensis*	0.55	0.25	0.24	0.09	0.32	0.17	0.17	0.26	0.31	0.25	0.28	0.25	0.33	0.12	0.12	0.24	0.25	0.26	0.24	0.27	0.24
**31**	*Cyrtodactylus thirakhupti*	0.52	0.22	0.24	0.08	0.31	0.17	0.19	0.22	0.28	0.23	0.26	0.22	0.31	0.10	0.13	0.21	0.22	0.23	0.21	0.23	0.24
**32**	*Cyrtodactylus linnoensis*	0.51	0.25	0.27	0.22	0.33	0.25	0.26	0.25	0.31	0.25	0.26	0.24	0.33	0.23	0.22	0.24	0.24	0.26	0.22	0.25	0.25
**33**	*Cyrtodactylus sadanensis*	0.49	0.23	0.24	0.21	0.31	0.23	0.24	0.23	0.30	0.23	0.22	0.21	0.32	0.21	0.20	0.22	0.21	0.23	0.22	0.23	0.23
**34**	*Cyrtodactylus yathepyanensis*	0.50	0.24	0.25	0.24	0.32	0.25	0.24	0.24	0.31	0.24	0.25	0.24	0.32	0.23	0.21	0.24	0.23	0.26	0.23	0.24	0.24
**35**	*Cyrtodactylus sadansinensis*	0.49	0.23	0.25	0.25	0.30	0.24	0.26	0.24	0.29	0.24	0.23	0.21	0.31	0.23	0.23	0.23	0.22	0.24	0.20	0.23	0.23
**36**	*Cyrtodactylus pharbaungensis*	0.50	0.22	0.25	0.24	0.29	0.25	0.27	0.22	0.28	0.21	0.23	0.21	0.29	0.23	0.24	0.23	0.21	0.23	0.19	0.22	0.21
**37**	*Cyrtodactylus sanpelensis*	0.49	0.22	0.23	0.23	0.28	0.23	0.25	0.23	0.29	0.23	0.23	0.21	0.29	0.22	0.22	0.22	0.21	0.23	0.19	0.22	0.22
**38**	*Cyrtodactylus linnwayensis*	0.53	0.26	0.30	0.28	0.30	0.28	0.29	0.25	0.29	0.27	0.28	0.26	0.31	0.28	0.26	0.27	0.24	0.25	0.25	0.26	0.25
**39**	*Cyrtodactylus shwetaungorum*	0.51	0.27	0.28	0.29	0.30	0.28	0.30	0.27	0.31	0.28	0.29	0.26	0.33	0.27	0.26	0.26	0.25	0.26	0.26	0.28	0.26
**40**	*Cyrtodactylus elok*	0.56	0.33	0.33	0.32	0.22	0.32	0.32	0.34	0.23	0.34	0.35	0.31	0.24	0.32	0.32	0.31	0.33	0.35	0.30	0.34	0.33
**41**	*Cyrtodactylus interdigitalis*	0.53	0.33	0.33	0.33	0.22	0.32	0.32	0.32	0.09	0.32	0.34	0.31	0.08	0.32	0.32	0.31	0.32	0.33	0.31	0.32	0.31

Table S1 (Continued) Interspecific uncorrected p-distance (percentage) based on the sequences of *ND2* gene (below the diagonal line) and intraspecific p-distance (on the diagonal line) of *Cyrtodactylus* species in phylogenetic analysis.

	**Species**	**21**	**22**	**23**	**24**	**25**	**26**	**27**	**28**	**29**	**30**	**31**	**32**	**33**	**34**	**35**	**36**	**37**	**38**	**39**	**40**	**41**
**25**	*Cyrtodactylus tigroides* (AP018116)	0.28	0.30	0.28	0.24	-																
**26**	*Cyrtodactylus lenya*	0.27	0.27	0.27	0.23	0.22	0.00															
**27**	*Cyrtodactylus oldhami* (captive)	0.26	0.27	0.25	0.22	0.14	0.21	-														
**28**	*Cyrtodactylus oldhami* (Phang-Nga)	0.26	0.28	0.28	0.21	0.11	0.20	0.12	-													
**29**	*Cyrtodactylus oldhami* (Ranong)	0.25	0.28	0.25	0.22	0.04	0.20	0.12	0.08	-												
**30**	*Cyrtodactylus payarhtanensis*	0.27	0.30	0.29	0.23	0.11	0.20	0.13	0.10	0.08	0.00											
**31**	*Cyrtodactylus thirakhupti*	0.25	0.25	0.26	0.21	0.10	0.18	0.12	0.08	0.08	0.09	0.01										
**32**	*Cyrtodactylus linnoensis*	0.27	0.25	0.26	0.24	0.25	0.25	0.24	0.23	0.24	0.23	0.22	0.00									
**33**	*Cyrtodactylus sadanensis*	0.24	0.23	0.23	0.20	0.24	0.23	0.23	0.21	0.22	0.21	0.20	0.08	0.00								
**34**	*Cyrtodactylus yathepyanensis*	0.25	0.25	0.26	0.23	0.25	0.25	0.25	0.23	0.24	0.24	0.23	0.11	0.10	0.01							
**35**	*Cyrtodactylus sadansinensis*	0.25	0.24	0.24	0.21	0.28	0.24	0.25	0.24	0.25	0.24	0.23	0.22	0.21	0.22	0.00						
**36**	*Cyrtodactylus pharbaungensis*	0.24	0.23	0.23	0.21	0.27	0.22	0.26	0.24	0.24	0.23	0.22	0.22	0.20	0.22	0.10	0.00					
**37**	*Cyrtodactylus sanpelensis*	0.25	0.24	0.23	0.20	0.24	0.21	0.24	0.22	0.22	0.22	0.21	0.22	0.19	0.21	0.10	0.06	0.00				
**38**	*Cyrtodactylus linnwayensis*	0.28	0.28	0.27	0.25	0.32	0.28	0.28	0.28	0.29	0.30	0.27	0.25	0.24	0.25	0.25	0.24	0.25	0.00			
**39**	*Cyrtodactylus shwetaungorum*	0.29	0.28	0.29	0.24	0.31	0.27	0.29	0.29	0.29	0.29	0.28	0.26	0.24	0.26	0.25	0.25	0.25	0.06	0.00		
**40**	*Cyrtodactylus elok*	0.36	0.33	0.34	0.29	0.36	0.32	0.33	0.32	0.33	0.33	0.32	0.34	0.33	0.34	0.33	0.30	0.32	0.31	0.32	-	
**41**	*Cyrtodactylus interdigitalis*	0.34	0.32	0.33	0.30	0.36	0.31	0.33	0.32	0.33	0.34	0.32	0.35	0.34	0.34	0.32	0.30	0.31	0.32	0.33	0.23	0.02
