# Supplementary material for: Phylogenetic analyses of distantly related clades of bent-toed geckos (genus Cyrtodactylus) reveal an unprecedented amount of cryptic diversity in northern and western Thailand
Source: Sci Rep. 2021 Jan 27;11:2328. doi: 10.1038/s41598-020-70640-8 (PMC7840752; doi:10.1038/s41598-020-70640-8)
Supplement: Supplementary file 2 — Supplementary file2 [file 41598_2020_70640_MOESM2_ESM.pdf]

Supplementary information
Table S2

Phylogenetic analyses of distantly related clades of bent-toed geckos (genus *Cyrtodactylus*) reveal an unprecedented amount of cryptic diversity in northern and western Thailand

Siriwadee Chomdej[1,2]*, Waranee Pradit[1], Chatmongkon Suwannapoom[3], Parinya Pawangkhanant[3], Korakot Nganvongpanit[4,5], Nikolay A. Poyarkov[6,7], Jing Che[8,9], Yangchun Gao[10], Shiping Gong[10]*

Table S2 List of specimens used for phylogenetic analysis. N/A: not available.

**Species**	**Localities**	**Vouchers**	**Accession Numbers**	**References**
Cyrtodactylus aequalis	Kyaiktiyo Hill, Mon State, Myanmar	LSUHC 12895	MF872275	Grismer, et al. [19]
Cyrtodactylus aequalis	Kyaiktiyo Hill, Mon State, Myanmar	LSUHC 14055	MN534918	Grismer, et al. [37]
Cyrtodactylus auribalteatus	N/A	N/A	AP018116	Areesirisuk, et al. [54]
Cyrtodactylus brevipalmatus	Khao Ramrome, Ron Phibun District, Nakhon Si Thammarat, Thailand	AUP-00573	MT468899	This study
Cyrtodactylus dammathetensis	Dammathet Cave 19.8 km east of Mawlamyine, Mawlamyine District, Mon State, Myanmar	LSUHC 12862	MF872276	Grismer, et al. [19]
Cyrtodactylus dammathetensis	Dammathet Cave 19.8 km east of Mawlamyine, Mawlamyine District, Mon State, Myanmar	LSUHC 12864	MF872278	Grismer, et al. [19]
Cyrtodactylus doisuthep	Doi Suthep, Mueang District, Chiang Mai Province, Thailand	AUP-00777	MT497801	This study
Cyrtodactylus dumnuii	cave near Doi Phrabart (formerly Tham (Cave) Phabartmaejon), Chiang Dao District, Chiang Mai Province, Thailand	AUP-00769	MT497802	This study
Cyrtodactylus dumnuii	Doi Phrabart abbey (formerly Tham (Cave) Phabartmaejon), Chiang Dao District, Chiang Mai Province, Thailand	AUP-00770	MT497803	This study
Cyrtodactylus elok	the Gap, Fraser's Hill, Pahang, West Malaysia	LSUHC 6471	JQ889180	Johnson, et al. [55]
Cyrtodactylus erythrops	Coral Cave, Pang Mapha District, Mae Hong Son Province, Thailand	AUP-00771	MT497806	This study
Cyrtodactylus interdigitalis	Nakai District, Khammouan Province, Laos	FMNH 255454	JQ889181	Johnson, et al. [55]
Cyrtodactylus inthanon	Tiger Head mountain, Doi Inthanon National Park, Chom Thong District, Chiang Mai Province, Thailand	AUP-00156	MT497800	This study
Cyrtodactylus lenya	the proposed Lenya National Park Extension, Tanintharyi Region, Myanmar	USNM 587788	KY041653	Connette, et al. [56]
Cyrtodactylus lenya	the proposed Lenya National Park Extension, Tanintharyi Region, Myanmar	USNM 587789	KY041652	Connette, et al. [56]
Cyrtodactylus lenya	the proposed Lenya National Park Extension, Tanintharyi Region, Myanmar	CAS 260233	KY041655	Connette, et al. [56]
Cyrtodactylus linnoensis	Linno Cave region 5 km south-west of Hpa-an, Hpa-an District, Kayin State, Myanmar	BYU 52230	MF872290	Grismer, et al. [19]

Table S2 (Continued) List of specimens used for phylogenetic analysis. N/A: not available.

**Species**	**Localities**	**Vouchers**	**Accession Numbers**	**References**
Cyrtodactylus linnoensis	Linno Cave region 5 km south-west of Hpa-an, Hpa-an District, Kayin State, Myanmar	LSUHC 12829	MF872297	Grismer, et al. [19]
Cyrtodactylus linnwayensis	Yum Twing Gyi Cave, Linn-Way Village, 12.7 km north-east of Ywangan, Taunggyi District, Shan State, Myanmar	BYU 52214	MF872280	Grismer, et al. [19]
Cyrtodactylus linnwayensis	Yum Twing Gyi Cave, Linn-Way Village, 12.7 km north-east of Ywangan, Taunggyi District, Shan State, Myanmar	LSUHC 12983	MF872287	Grismer, et al. [19]
Cyrtodactylus oldhami	captive	JB 126	JX440548	Wood, et al. [18]
Cyrtodactylus oldhami	Kraburi District, Phang-Nga Province, Thailand	MS 460	MF872301	Grismer, et al. [19]
Cyrtodactylus oldhami	Muang District, Ranong Province, Thailand	MS 585	MF872302	Grismer, et al. [19]
Cyrtodactylus payarhtanensis	the proposed Lenya National Park, Tanintharyi Region, Myanmar	USNM 587791	KY041658	Connette, et al. [56]
Cyrtodactylus payarhtanensis	the proposed Lenya National Park, Tanintharyi Region, Myanmar	USNM 587408	KY041654	Connette, et al. [56]
Cyrtodactylus pharbaungensis	Pharpoun Cave 38.4 km south-east of Mawlamyine, Mawlamyine District, Mon State, Myanmar	BYU 52215	MF872303	Grismer, et al. [19]
Cyrtodactylus pharbaungensis	Pharpoun Cave 38.4 km south-east of Mawlamyine, Mawlamyine District, Mon State, Myanmar	LSUHC 12870	MF872304	Grismer, et al. [19]
Cyrtodactylus sadanensis	Sadan Cave 17 km south-east of Hpa-an, Hpa-an District, Kayin State, Myanmar	BYU 52216	MF872310	Grismer, et al. [19]
Cyrtodactylus sadanensis	Sadan Cave 17 km south-east of Hpa-an, Hpa-an District, Kayin State, Myanmar	LSUHC 12848	MF872322	Grismer, et al. [19]
Cyrtodactylus sadansinensis	Sadan Sin Cave 10.5 km north-west of Mawlamyine, Mawlamyine District, Mon State, Myanmar	BYU 52220	MF872325	Grismer, et al. [19]
Cyrtodactylus sadansinensis	Sadan Sin Cave 10.5 km north-west of Mawlamyine, Mawlamyine District, Mon State, Myanmar	LSUHC 12859	MF872330	Grismer, et al. [19]
Cyrtodactylus saiyok	Suan Phueng District, Ratchaburi Province, Thailand	AUP-00668	MT468900	This study

Table S2 (Continued) List of specimens used for phylogenetic analysis. N/A: not available.

Species	Localities	Vouchers	Accession Numbers	References
Cyrtodactylus saiyok	Sai Yok District, Kanchanaburi Province, Thailand	AUP-00773	MT497805	This study
Cyrtodactylus saiyok	Sai Yok National Park, Kanchanaburi Province, Thailand	MS 484	MF872308	Grismer, et al. [19]
Cyrtodactylus saiyok	Suang Phung, Ratchaburi Province, Thailand	MS 480	MF872309	Grismer, et al. [19]
Cyrtodactylus sanook	Tham Sanook, Muang District, Chumphon Province, Thailand	AUP-00570	MT468898	This study
Cyrtodactylus sanpelensis	Sanpel Cave 21.3 km south-east of Mawlamyine, Mawlamyine District, Mon State, Myanmar	BYU 52224	MF872334	Grismer, et al. [19]
Cyrtodactylus sanpelensis	Sanpel Cave 21.3 km south-east of Mawlamyine, Mawlamyine District, Mon State, Myanmar (N16°22.427,E97°46.388)	LSUHC 12877	MF872337	Grismer, et al. [19]
Cyrtodactylus shwetaungorum	5.3 km north of Pyinyaung Village at the Apache Cement factory mining site, Mandalay Region	BYU 52227	MF872348	Grismer, et al. [19]
Cyrtodactylus shwetaungorum	5.3 km north of Pyinyaung Village at the Apache Cement factory mining site, Mandalay Region	LSUHC 12898	MF872353	Grismer, et al. [19]
Cyrtodactylus sinyineensis	Sin Yine Cave 18.5 km south-east of Hpa-an, Hpa-an District, Kayin State, Myanmar	LSUHC 12835	MF872354	Grismer, et al. [19]
Cyrtodactylus sinyineensis	Sin Yine Cave 18.5 km south-east of Hpa-an, Hpa-an District, Kayin State, Myanmar	LSUHC 12836	MF872355	Grismer, et al. [19]
Cyrtodactylus sinyineensis	Sin Yine Cave 18.5 km south-east of Hpa-an, Hpa-an District, Kayin State, Myanmar	LSUHC 12837	MF872356	Grismer, et al. [19]
Cyrtodactylus sp.	Phu Rua District, Loei Province, Thailand	FMNH 265806	JX519471	Grismer, et al. [14]
Cyrtodactylus sp. 1	Chao Doi Waterfall, Mae Moei National Park, Tha Song Yang District, Tak Province, Thailand	AUP-00683	MT468903	This study

Table S2 (Continued) List of specimens used for phylogenetic analysis. N/A: not available.

**Species**	**Localities**	**Vouchers**	**Accession Numbers**	**References**
Cyrtodactylus sp. 1	Chao Doi Waterfall, Mae Moei National Park, Tha Song Yang District, Tak Province, Thailand	AUP-00684	MT468904	This study
Cyrtodactylus sp. 1	Mae Moei National Park, Tha Song Yang District, Tak Province, Thailand	AUP-00685	MT468905	This study
Cyrtodactylus sp. 1	Mae Moei National Park, Tha Song Yang District, Tak Province, Thailand	AUP-00687	MT468906	This study
Cyrtodactylus sp. 2	Mae Usu Cave, Mae Moei National Park, Tha Song Yang District, Tak Province, Thailand	AUP-00673	MT468901	This study
Cyrtodactylus sp. 3	Sri Fah Cave, Mae Sot District, Tak Province, Thailand	AUP-00692	MT468912	This study
Cyrtodactylus sp. 4	Mae Wong National Park, Khlong Lan District, Kamphaeng Phet Province, Thailand	AUP-00775	MT497807	This study
Cyrtodactylus sp. 5	Mae Klang Luang, Doi Inthanon National Park, Chom Thong District, Chiang Mai Province, Thailand	AUP-01027	MT468907	This study
Cyrtodactylus sp. 6	Mae Hong Son Bamboo Complex, Mueang District, Mae Hong Son Province, Thailand	AUP-01576	MT468908	This study
Cyrtodactylus sp. 7	Thong Pha Phum National Park, Thong Pha Phum District, Kanchanaburi Province, Thailand	AUP-01718	MT468910	This study
Cyrtodactylus sp. 8	Near Vajiralongkorn dam, Thong Pha Phum National Park, Thong Pha Phum District, Kanchanaburi Province, Thailand	AUP-01722	MT468911	This study
Cyrtodactylus sp. 9	Thong Pha Phum National Park, Thong Pha Phum District, Kanchanaburi Province, Thailand	AUP-01715	MT468909	This study
Cyrtodactylus sp. 10	Chao Doi Waterfall, Mae Moei National Park, Tha Song Yang District, Tak Province, Thailand	AUP-00680	MT468902	This study
Cyrtodactylus thirakhupti	Tham Khao Sonk Hill, Surat Thani Province, Thailand	ZMKU_R_00732, LSUHC 12467	MF872357	Grismer, et al. [19]
Cyrtodactylus thirakhupti	Tham Khao Sonk Hill, Surat Thani Province, Thailand	ZMKU_R_00733, LSUHC 12468	MF872358	Grismer, et al. [19]

Table S2 (Continued) List of specimens used for phylogenetic analysis. N/A: not available.

Species	Localities	Vouchers	Accession Numbers	References
Cyrtodactylus thirakhupti	N/A	N/A	AP018115	Areesirisuk, et al. [54]
Cyrtodactylus tigroides	Wang Krachae Subdistrict, Sai Yok District, Kanchanaburi Province, Thailand	AUP-00776	MT497804	This study
Cyrtodactylus tigroides	Ban Tha Sao, Sai Yok District, Kanchanaburi Province, Thailand	IRSNB 2380	JX440562	Wood, et al. [18]
Cyrtodactylus tigroides	N/A	N/A	AP018118	Areesirisuk, et al. [54]
Cyrtodactylus welpyanensis	Wel Pyan Cave 35 km north of Hpa-an, Hpa-an District, Kayin State, Myanmar	LSUHC 12785	MF872360	Grismer, et al. [19]
Cyrtodactylus welpyanensis	Wel Pyan Cave 35 km north of Hpa-an, Hpa-an District, Kayin State, Myanmar	LSUHC 12792	MF872362	Grismer, et al. [19]
Cyrtodactylus yathepyanensis	Yathe Pyan Cave 9 km south-west of Hpa-an, Hpa-an District, Kayin State, Myanmar	BYU 52229	MF872364	Grismer, et al. [19]
Cyrtodactylus yathepyanensis	Yathe Pyan Cave 9 km south-west of Hpa-an, Hpa-an District, Kayin State, Myanmar	LSUHC 12823	MF872367	Grismer, et al. [19]
**Outgroup**				
Gekko gecko	Shwesettaw wildlife sanctuary, Mimbu Township, Magway Division, Myanmar	CAS 213628	JN019053	Rösler, et al. [57]
Ptychozoon kaengkrachanense	Thong Pha Phum National Park, Thong Pha Phum District, Kanchanaburi Province, Thailand	AUP-01710	MT468895	This study
Dixonius siamensis	Thong Pha Phum National Park, Thong Pha Phum District, Kanchanaburi Province, Thailand	AUP-01724	MT468896	This study
Hemidactylus frenatus	Rathegala, Sri Lanka	AMB 7420	EU268359	Bauer, et al. [58]
